# Mental Health among Italian Children and Adolescents during and after the SARS-CoV-2 Pandemic: A Professionals’ Focus Group Study

**DOI:** 10.3390/jcm12134270

**Published:** 2023-06-26

**Authors:** Maria Luisa Scattoni, Martina Micai, Angela Caruso, Letizia Gila, Francesca Fulceri, Giulia Galati, Maria Antonella Costantino, Massimo Molteni, Paolo Petralia, Marcello Lanari, Cristiana Corinaldesi, Carla Garlatti

**Affiliations:** 1 Research Coordination and Support Service, Istituto Superiore di Sanità, Viale Regina Elena 299, 00161 Rome, Italy; martina.micai@iss.it (M.M.); angela.caruso@iss.it (A.C.); letizia.gila@iss.it (L.G.); francesca.fulceri@iss.it (F.F.); giulia.galati@iss.it (G.G.); 2 Child and Adolescent Neuropsychiatric Unit, Foundation IRCCS Cà Granda Ospedale Maggiore Policlinico, 20122 Milan, Italy; antonella.costantino@policlinico.mi.it; 3 Scientific Institute IRCSS E. Medea, Unit of Child Psychopathology, 23842 Bosisio Parini, Italy; massimo.molteni@lanostrafamiglia.it; 4 ASL4 SSR Liguria, 16043 Chiavari, Italy; ppetralia65@gmail.com; 5 DISSAL Department of Health Sciences, University of Genoa, 16100 Genoa, Italy; 6 Pediatric Emergency Unit, IRCCS Azienda Ospedaliera Universitaria di Bologna, 40138 Bologna, Italy; marcello.lanari@unibo.it; 7 Italian Ombudsperson for Children and Adolescents, 00196 Rome, Italy; corinaldesi@garanteinfanzia.org (C.C.); garlatti@garanteinfanzia.org (C.G.)

**Keywords:** mental health, child, adolescent, SARS-CoV-2 pandemic, services, Italy, focus groups

## Abstract

The SARS-CoV-2 pandemic had a negative impact on the mental health of children and adolescents. Eight focus groups and six individual hearings were conducted to gain insights from the perspectives of 97 Italian professionals from a variety of disciplines, including child and adolescent neuropsychiatrists, pediatricians, general practitioners, social workers, psychologists, teachers, school deans, non-governmental organizations, and a researcher. Urban and rural settings were represented. The present research has been promoted by the Italian Independent Authority for Children and Adolescents (Autorità Garante per l’Infanzia e l’Adolescenza, AGIA) in collaboration with the National Institute of Health (Istituto Superiore di Sanità, ISS) and the Ministry of Education and its scientific value has been supervised by a Scientific Committee. The results of the focus groups’ discussions revealed increased vulnerability, with the onset of new mental health disorders in healthy individuals and those in a condition of disadvantage, migratory contexts, and with disabilities. The already critical pre-pandemic structural and operational issues of existing services have been exacerbated. In healthcare, the activation of telemedicine has been a great asset but at the same time has generated challenges and critical issues that are still unresolved. Professionals emphasized the need to pay special attention to planning integrated responses aimed at overcoming inequalities and fragmentation. The result of this initiative translates into a set of operational recommendations useful for guiding investments and policies directed toward the protection of the mental health of minors in the health, educational, and social spheres from the outset.

## 1. Introduction

The SARS-CoV-2 pandemic and containment measures implemented to address it have had and are expected to continue having significant effects on people’s lives, particularly on the well-being of young individuals. Numerous studies have examined the impact of the pandemic on the mental health of children and youth [1,2,3,4,5,6,7,8,9,10,11]. Although more longitudinal studies are needed and sampling bias is present [9], the most frequently reported disorders in the literature are depression, anxiety, sleep disorder, post-traumatic stress disorder, and emotional and behavioral disorders [12,13,14]. The pandemic may also have resulted in an increase in eating disorders [15] and rates of suicide, suicidal ideation, and self-harm [16]. Some authors have reported the increased use of alcohol, cannabis, tobacco, and internet gaming in adolescents [17,18,19,20]. Although the moderate, purpose-driven increase in technology use has been adaptive to the demands of the pandemic period, excessive involvement and/or exposure to specific online activities—such as gambling, viewing pornographic material, video games, social media use, and shopping—may have led to the most vulnerable individuals experiencing serious problems and an increased risk of disordered use or addiction [21].

Most studies conducted so far have explored the consequences of the SARS-CoV-2 pandemic only in the short term and with significant methodological biases [5]. There is evidence that the quality and magnitude of the impact on neurodevelopment and mental health of children and adolescents are influenced by various factors such as age, educational status, pre-existing mental health conditions, being economically disadvantaged, being/having been in forced isolation, or having social support [22,23,24,25]. In this regard, a recent study on predictors of poor outcomes emphasized the role of social isolation, duration of exposure to screens and excessive use of social media, parental stress, parent–child relationship issues, low socioeconomic status, and pre-existing mental health conditions and/or disabilities [26]. Further studies, conducted in different areas of Italy, confirmed that the pandemic and measures put in place to contain it may have had a major impact on the neurodevelopment and mental health of children and youth, especially for those who were already living in more disadvantaged conditions [27,28,29,30,31]. In this context, numerous alarm signals have been received from the territorial and hospital garrisons for the reception and care of underaged persons—pediatric emergency rooms, outpatient clinics, social services, territorial and hospital services of child and adolescent neuropsychiatry—which have recorded an increase in cases of distress, self-harm, eating and sleeping disorders, alcohol or drug addictions, a sense of loneliness, and social withdrawal, especially with reference to adolescents.

The aim of the present research is to investigate the effects of the SARS-CoV-2 pandemic on the neurodevelopment and mental health of underaged people and the effects that government measures that have been implemented to contain the emergency have brought about. This research delves into the impact of the pandemic on a few specific groups: (1) those who did not suffer from mental health problems prior to the pandemic to check for their onset; (2) children and youth with pre-existing neuropsychiatric disorders or vulnerabilities who may have experienced their exacerbation; and (3) children and youth with disabilities or severe neuropsychiatric disorders who experienced the disruption or limited functioning of therapeutic activities and interventions during the pandemic. The present research has been promoted by the Italian Independent Authority for Children and Adolescents (Autorità Garante per l’Infanzia e l’Adolescenza, AGIA) and coordinated by the National Institute of Health (Istituto Superiore di Sanità, ISS) in collaboration with the Ministry of Education, and two research and clinical centers of excellence (IRCCS Ca’ Granda Policlinico and IRCCS Medea—Bosisio Parini). AGIA also appointed a Scientific Committee, including representatives of the Italian Pediatric Scientific Society, Child and Adolescent Neuropsychiatric Society, Psychological Scientific Society, National Council of the Order of Social Workers, and three experts in the field of child and adolescent mental health, with the function of advising and planning activities involving children and adolescents, as well as integrating the various phases and different components involved in the research.

## 2. Materials and Methods

**Figure 1:**
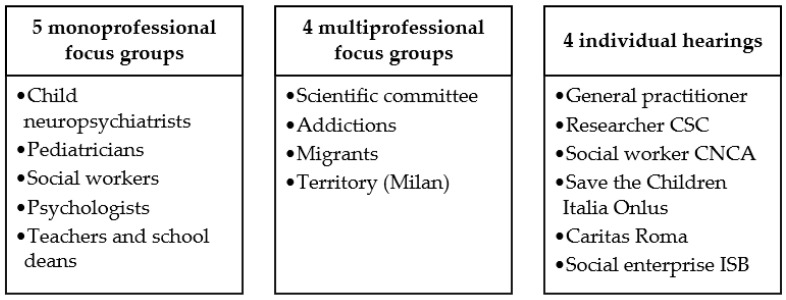
Summary of the focus groups and individual hearings.

The Scientific Committee (one Chair, the Chair and an elected councilor of the National Council of the Order of Social Workers, the Chair and a representative of the National Council Order psychologists, the Chair of the Italian Society of Pediatrics, the Vice-President of the Italian Society of Child and Adolescent Neuropsychiatry, and three experts in the field of child and adolescent mental health) was asked to indicate experts pertaining to the professional field according to the following criteria: (1) professionals from the north, central, south, and island areas of Italy; (2) professionals dealing with minors of different ages; (3) professionals working in different delivery settings such as emergency departments, hospitals, social services, territorial services, counseling, and child and adolescent neuropsychiatry. Professionals selected from the field of child and adolescence neuropsychiatrists/psychiatrists, psychologists, and social workers included those with specific experience and roles in the following areas: disabilities, addictions, eating disorders, migrants/foreigners, and other frailties. Professionals from rural and urban settings were selected. Monoprofessional focus groups involved experts from the same professional background, albeit from different parts of the country and different organizational settings. Multiprofessional focus groups involved heterogeneous professionals who shared a particular characteristic in working with children and youth (e.g., addictions, migrants/foreigners). The following interviews were conducted between August 2021 and February 2022: (1) five monoprofessional focus groups involving, separately, child and adolescent neuropsychiatrists, pediatricians, social workers, psychologists, teachers, and school leaders; (2) four multiprofessional focus groups: one focused on the topic of pathological addictions; another delved into the impact of the pandemic on unaccompanied foreign minors; a third, having territorial characteristics, involved professionals who work in contact with underage persons in the Milan area (because of the complexity of the large metropolitan context in the pandemic era); and a fourth involved the members of the Scientific Committee. Each focus group generally included between 8 and 15 professionals; (3) six individual hearings, directed at specific professionals or private social organizations: a general practitioner, a researcher from the Codici Social Cooperative (Cooperativa Sociale Codici, CSC), a social worker from the National Coordination of Shelter Communities (Coordinamento Nazionale Comunità di Accoglienza, CNCA), a representative from Save the Children Italia Onlus, a representative from Caritas Roma, and a representative from Social Enterprise with Children (Impresa Sociale con i Bambini, ISB). The selection of monoprofessional and multiprofessional groups, along with individual hearings, involves professionals responsible for working with minors. Monoprofessional groups focus on specific issues, allowing experts to contribute their unique perspectives. Multiprofessional groups promote collaboration and a holistic approach. Individual hearings enable professionals to share firsthand experiences and provide personalized insights from their professional point of view. A total of 97 Italian professionals (age range 30 to 50 years old, 66% females) from a variety of disciplines were involved. Figure 1 is a summary of the focus groups and individual hearings held.

Three-hour focus groups were conducted jointly by a representative of the AGIA and ISS, as well as a member of the Scientific Committee or another professional selected by the Committee. All focus groups and individual hearings were conducted remotely using the Microsoft Teams platform. Only one focus group, the territorial focus group, was conducted in Milan.

The group discussion was introduced by a description of the research project by the presenters, which was followed by a request for a brief introduction from all participants with a virtual table turn. Participants were asked to specify: their name, affiliation, geographic area, specific professional field, and prevalent age group of minors with whom they work. The research team of the ISS transcribed the relevant reports, which were then shared with the participants in the same focus groups for comments and additions, and with the Scientific Committee.

**Figure 2:**
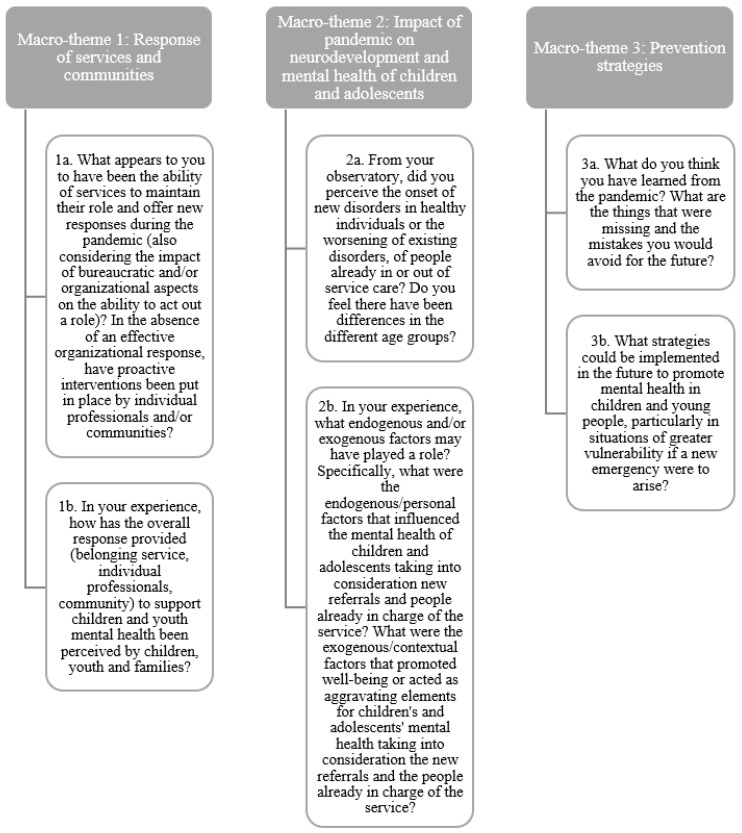
Macro-themes proposed to the participants of the focus groups.

The dual-moderator focus groups were conducted with a low level of directivity to achieve authentic interaction and exchange among members. The moderators proposed three discussion themes to the groups (Figure 2), asking the participants to address them with the four phases of the pandemic in mind: phase 1, coinciding with the total lockdown (in Italy from February 2020 to June 2020); phase 2, referring to the summer of 2020 when several activities had reopened; phase 3, referring to the second wave of SARS-CoV-2 pandemic (in Italy, from September to December 2020); and, finally, phase 4, between January and November 2021 (the time when the first phase of the survey was conducted, which corresponded to the period of the gradual reopening of activities). After the introduction and reading of the themes, the interventions were spontaneous, and the moderator intervened only to lead back to the focus topic and ensure maximum participation of each member. One moderator was in charge of asking the questions and the other to make sure the questions were answered. Taking into account the purpose of the focus groups, specific areas of discussion were identified: (1) perceptions of the onset of new disorders in healthy individuals or worsening of existing disorders; (2) role played by various exogenous and endogenous factors as elements of vulnerability: age, geography, socioeconomic conditions, social isolation, presence of neurodevelopmental or psychiatric disorders; (3) perceptions of factors supporting the resilience of children and families; (4) role of the service network and community. These items were summarized into three macro-themes proposed for discussion by focus participants: (1) service and community response; (2) impact of the pandemic on the neurodevelopment and mental health of children and young people; and (3) prevention strategies. Figure 2 shows the questions posed in relation to the three macro-themes identified.

An email describing the three macro-themes was sent to each focus group member a few days before the focus group was to be held. It was appropriate to favor reflection by each professional, rather than an extemporaneous response, given the sensitivity and complexity of the issues posed. Participants’ discussion was not compromised by prior knowledge of the issues, which, instead, were well explored and the subject of an articulate and participatory debate.

## 3. Results

Below are the main findings from the discussions of the nine focus groups and six individual expert hearings. The results are reported for three macro-themes in the same way that they were posed and addressed by the participants. Thematic insights related to schools, addictions, and migrants are also reported. It was possible to highlight the commonalities and divergences that emerged among the various discussions, as well as the impact in terms of the response they had on the neurodevelopment and mental health of children and youth. The general attitude of each group was one of active participation and enthusiasm. Instructions were generally well received. The three macro-themes all had extensive participation. All groups actively participated by contributing to their specific professional field.

### 3.1. Macro-Theme 1: Response of Services and Communities

The first macro-topic proposed to the groups concerned the responsiveness of services and communities to the needs of children and adolescents during the pandemic and the related perceptions of the underaged people attending them and their families. Professionals pointed to a highly variable situation based on the type of service and context of origin (e.g., hospital vs. territorial, urban vs. rural, social vs. health), the geographical area (north, central, south, and island areas of Italy)—also in relation to the variable exacerbation of the pandemic in the different territories—as well as the institutional and regulatory context in terms of specific regional or local agency directives. Lack of services at the territorial level, poor synergy, or inadequate human resources also resulted in inhomogeneous responses. The first emerging finding relates to the inequalities of the emergency responses related to local territorial, institutional, and organizational contexts. In general, in all focus groups, respondents reported that the critical issues in services, which were pre-existing to the pandemic, were greatly exacerbated in the pandemic phase. Therefore, in relation to the increase in demand, there is evidence of a predominantly inadequate, often disorganized, and improvised response by healthcare, educational, and social services.

In general, during the pandemic period, children and families who were afferent to healthcare and social services experienced difficulties in identifying and contacting the professionals they needed due to insufficient communication of the new modified access modalities for the pandemic or prolonged closure of some of the services. As a result, needs have not been met and often families, at least those whose socioeconomic conditions allow it, have turned, and still turn to private services and professionals. The pandemic and measures to contain it have created a polarization between those who have remained or have been able to remain hooked to the service system, benefiting from the support interventions, and those who, on the contrary, have moved away or have had no way to access services, finding themselves facing solitude and challenging living conditions that have become even more critical and have resulted in a further increased risk to neurodevelopment and mental health.

Participants also emphasized the lack of specific attention to neurodevelopment and children’s and adolescents’ mental health by policymakers during the pandemic crisis, as well as the absence of a “strategic direction”, at the national or regional level, for the management of the increased demand for support from minors and their families, including in terms of preventive actions and the promotion of neurodevelopment and mental health. One participant said: “The pandemic has highlighted that we have dwelt too much on the more medical concept of health and too little on mental health.” The legislative powers of the Italian Regions and Autonomous Provinces during the pandemic, as well as the discretion of their responses in a quantitative and/or qualitative sense, have left citizens disoriented.

The main organizational innovation activated by the services to promptly respond to the mental health needs of children and adolescents in the pandemic phase concerns telemedicine.

In the initial phase of the pandemic, the use of digital tools for professional purposes was adopted based on voluntary initiatives to ensure the continuity of therapeutic, rehabilitative, educational, and social activities for children and youth who already under the care of services, rather than for handling new requests for help or answering diagnostic questions.

Later, regional directives allowed for the formal use of telemedicine interventions, and professionals were able to consolidate the use of technological tools, albeit with extreme inhomogeneity, throughout the country. The use of IT tools increased in frequency and improved the quality of the relationship between professionals, services, and users, as well as the investment in distance learning initiatives, with positive impact and perception. Although professionals reported a strong concern about cybersecurity, confidentiality, and privacy in the use of IT tools, telemedicine proved to be an extremely important resource. Many professionals decided to maintain the telemedicine modality in the post-pandemic period, especially for certain activities and/or in situations of necessity (e.g., for patients who live far from the clinical center, or to facilitate the networking of services). Table 1 summarizes the perceptions, experiences, and factors that are considered negative and those considered positive and to be enhanced following the pandemic for the first macro-theme.

### 3.2. Macro-Theme 2: Impact of Pandemic on Neurodevelopment and Mental Health of Children and Adolescents

The second macro-theme proposed to the groups concerns the impact that the pandemic has had on the neurodevelopment and mental health of children and adolescents, with particular reference to both those who, before the SARS-CoV-2 pandemic, did not manifest neurodevelopment or psychiatric conditions and those who had previous conditions.

In the first pandemic phase, there was a “freeze” of requests for help, while from the summer of 2020 onward there was an increase in cases of mental distress and need for support. Professionals reported that, from the second wave onward, there was an increase in requests for help for the onset of all neuropsychiatric disorders or aggravation of previous situations in individuals already known to services, as also shown by emergency room admissions and/or hospitalization requests. Requests for support were made by parents, but also by adolescents themselves. In general, the professionals concur that there have been numerous cases of the worsening of pre-existing conditions in children and adolescents already in care, as well as the onset of new conditions, especially in subjects in conditions of vulnerability related to family, environmental, sociocultural, and economic conditions, and migratory backgrounds. The most frequently reported disorders and/or symptoms were: eating behavior disorders, suicidal ideation (attempted suicide and suicide), episodes of self-harm, sleep–wake rhythm alterations, and social withdrawal. It has also been pointed out that the onset of conditions or their worsening varies according to the age and stage of the pandemic. The most severely affected seem to be pre-adolescents and adolescents, especially those who are in the middle of school transitions, i.e., children who are about to start the first class of secondary school and the first year of college.

Professionals also recorded numerous frailties at the family level. The general increase in adult stress over worsening working conditions or job loss may have changed intergenerational dynamics, causing many parents to discover that they are more fragile and exposed than their daughters and sons were. There has also been a worsening of situations of domestic and witnessed violence, parental conflict, or loneliness. In some contexts, there has also been an apparent decrease in cases of abuse and violence (verbal, physical, and witnessed) on minors since the reduction of in-presence activities has made violence less visible and interceptable. On this aspect, one participant said: “The pandemic teaches that environmental factors are crucial in children’s neurodevelopment: without a quota of social contact, damage occurs.” Table 2 summarizes the perceptions, experiences, and factors that are negative and those that are positive and to be enhanced as a result of the pandemic for the second macro-theme.

The research also investigated the influence of endogenous and exogenous risk and resilience factors on the psycho-physical well-being of younger people (Table 3).

### 3.3. Macro-Theme 3: Prevention Strategies

The last item covered dealt with possible strategies for the promotion of neurodevelopment and mental health of children and young people, especially regarding situations of greater fragility, as well as measures to be put in place both to reduce their negative impact and achieve appropriate organizational responses in terms of prevention and in the event of a future emergency. All the professionals agreed on the importance of stimulating and strengthening the bio-psycho-social growth model of children and young people, to ensure a harmonious and global development and prevent discomfort and psychic suffering as much as possible. Proposals emerged regarding the implementation of homogeneous responses throughout the national territory, aimed at overcoming the regional fragmentation and disparities of social health services.

According to the participants, the pandemic has emphasized the need to “network” among existing services and implement stable working groups. In support of this view, one participant said: “We need to network to provide creative and timely responses.” To this end, it is necessary to organize activities and services in a well-structured and coordinated network among all systems dealing with childhood, adolescence, and family–including schools and the third sector—through strategic and cross-sectoral action. It seems fundamental to adopt a comprehensive overview that, at the same time, provides for interconnected and synergistic local interventions. Coordination at the territorial level of activities and services has to take place in a stable and continuous manner. The good practices implemented in some regional realities could be seconded and incorporated into all systems dealing with child and adolescent health at the national level, in a logic of synergy, mutual subsidiarity, and strengthening of the voluntary and non-profit sector realities, which are already widespread.

In addition to a more effective development of organizational aspects, the strengthening of existing structures—in terms of human and material resources—and the improvement in the connection and coordination among them was identified as a strategic action. An increase in health and social workers with specific skills and training in the management of neuropsychic difficulties and disorders has been considered necessary, as well as an increase in the staffing of child and adolescent neuropsychiatric community care services and the number of child and adolescent neuropsychiatric inpatient beds (whose number is very limited in Italy, with only 394 beds nationwide). One participant reported: “The pandemic has taught the need for a stable working group to deal with the difficulty and have a broader eye for observation.” Fundamental, moreover, is a greater redifferentiation of pathways based on a stepped care perspective, identifying low, medium, and high-intensity care pathways on the basis of users’ needs and implementing formalized linkages between facilities that guarantee the different types of pathways.

The presence of multidisciplinary teams appears to be essential to ensure holistic patient care, particularly for medium- or high-complexity users. A case manager is indicated for the latter, as a key figure to improve pathways of patient intake and care. Another important element is the ongoing training of operators, aimed at the development and maintenance of specific clinical competencies, the acquisition and enhancement of skills for the analysis and management of priorities, as well as the expansion and definition of interinstitutional networks and collaborative practices for outcome evaluation. Additionally, it was indicated as a strategic action to provide professionals with support for work and organizational well-being and burn-out prevention on an ongoing basis.

In all realities, especially in the most socio-economically and culturally disadvantaged ones, it has been considered necessary to provide actions to support parenting, distribute informational materials, and offer parent training paths to activate parenting skills in programs of welcoming and accompanying parenting, starting from birth. It has also been indicated that there is a need to create listening spaces and meeting places for collaboration between parenting couples. It is suggested to provide for the strengthening of strategies that increase awareness and enhance the relationship with expressive skills, relationship with the self and others, and the enhancement of approaches centered on the arts and natural world, giving rise to paths of outdoor education. In this sense, it is necessary to enhance spaces of integration and socialization for children and adolescents, spaces where they can express themselves and share, and activate personal and interpersonal resilience strategies, ensuring health-related or residential interventions. Indeed, the integration of fragile individuals into social contexts remains a primary goal; in this sense, increasing awareness and the use of advocacy tools by professionals, redesigning interventions with the involvement of the minors themselves, is identified as an action for improvement. In addition, to improve the quality of care for at-risk populations, such as children and youth with a migration history, the implementation of more standardized and uniform procedures is specifically mentioned as necessary, through, for example, a memorandum of understanding with receiving facilities. Table 4 summarizes the prevention strategies suggested by professionals.

### 3.4. Thematic Insights: Prevention Strategies for Schools, Unaccompanied Foreign Minors, and Additions

Professionals emphasized the need to give the school a central role, not only in terms of education and training, but also in promoting and monitoring the neurodevelopment and mental health of children and adolescents. The school, in fact, is one of the main places where young people grow up and build their own identity. It is also the place where they spend a lot of time, often more than at home, and where physiological discomforts, mental health problems, and neurodevelopmental disorders can be easily identified, even in a prevention logic.

The exchange of opinions that emerged during the focus groups allowed concrete actions to be suggested to make the educational context more proactive in supporting the prevention and identification of students’ discomforts. In particular, professionals suggest dedicating greater investments in schools on the subject of neurodevelopment and mental health in a systemic way and not just through projects, targeting especially peripheral areas and the most vulnerable students, taking a closer look at the economic, social, and cultural vulnerabilities of families.

Many professionals have also reported the need to include professional figures, such as psychologists and psycho-pedagogists in all schools who can provide support not only to students but also to the teaching staff, and in any case develop closer links with public territorial services to which such professionals refer. On this line, one participant said: “We need presence of school psychologists, processing spaces of what has happened, how this event is within the history of each of us, contact spaces where we feel safe and can have awareness of our resources.” The continuous training of teachers should be incentivized in order to enhance the competent use of digital tools, as well as to overcome traditional teaching methods.

The educational alliance between teachers and students, among teachers themselves, among students, and between the school and family, should be strengthened by valuing the tool of educational agreements in order to avoid them being reduced to a mere list of offers. One participant reported: “Comprehensive prevention actions are needed. Schools must lower the pressure on school performance and accommodate post-pandemic discomfort.” This goal can be achieved through the creation of workgroups and workshops, through the involvement of teachers, parents, underage people, transcultural operators, artists, sports operators, and stakeholders, with the aim to design extracurricular activities in artistic, musical, sports, and leisure fields.

To improve the quality of care for children and young people with a migration history, the need to develop more homogeneous responses and shared reference procedures has been specifically indicated, in addition to a memorandum of understanding with reception facilities. One participant reported: “Children can make us discover the value of migration; it is an opportunity to overcome with a different spirit the difficulties that may arise. [...] Sometimes it is possible to improve the response in the long run by changing some contextual conditions.”

The interviewed professionals consider the prevention of addictions to be of fundamental importance and that it should be increasingly closely linked to the promotion of neurodevelopment and mental health more generally, and not conducted in isolation. They also believe that funds and research dedicated to both pathological addictions and mental health should be implemented. Table 5 summarizes the prevention strategies suggested by professionals for thematic insights.

## 4. Discussion

The aim of this research is to investigate the impact of the SARS-CoV-2 pandemic on the neurodevelopment and mental health of minors, as well as the effects of government measures implemented to control the emergency. As part of the present research, more than 90 professionals who worked with children and adolescents in the pandemic phase in support of neurodevelopment and mental health were heard through focus groups and individual hearings. The focus groups and individual hearings brought out recurring aspects and themes, suggesting the possible implementation of different system strategies already in the immediate present. The result of this initiative translates into a set of operational recommendations useful for guiding, from the outset, investments and policies directed toward the protection of the mental health of minors in the social, health, and educational spheres. Beginning with the AGIA’s mission, the purpose of our activity has been to give precise guidance to policymakers to rearrange social and health policy choices in favor of minors and their families.

The professionals interviewed emphasized that the pandemic and measures implemented to contain it have had a considerable impact on the lives of children and their families, leading to a general sense of uncertainty and disorientation throughout the population. In children and adolescents in particular, the pandemic has resulted in a growing set of frailties, such as the exacerbation of already diagnosed neurodevelopmental and mental health conditions and the onset of disorders in vulnerable individuals (e.g., minors placed in socio-cultural disenfranchisement, migration, with disabilities, or other vulnerabilities), or in subjects who were healthy [32]. Professionals have witnessed a true “mental health emergency” [33] due to the continuous increase in demands in this area. The conditions most frequently reported by health professionals in adolescents were sleep–wake rhythm disturbances, emotional dysregulation, eating disorders, suicidal ideation and attempts, self-harm, and social withdrawal, but also a high sense of frustration and uncertainty, difficulties in cognitive and metacognitive regulation, as well as concentration difficulties, which generated school defaults and increased dropouts. The network of child and adolescent neuropsychiatry and developmental rehabilitation services, counseling and psychological services, and educational and social services in Italy’s various territorial realities has found itself responding to the considerable increase in requests from a situation of generalized lack of resources and high inhomogeneity in the pre-pandemic organization, resulting in inadequate and inequitable responses at the regional and local levels. The structural and operational criticalities of public and accredited services that existed before the pandemic (e.g., shortage of staff, economic resources, and training of professionals) were exacerbated during the emergency scenario.

In the health and social health field, the activation of telemedicine has been a great resource but, at the same time, has generated challenges and even inequalities that are still unresolved [34]. During the pandemic, activities carried out remotely offered important opportunities to give support to underage persons and families living far from clinical centers, to remodel health and social welfare activities in some cases, to schedule training for users and their families, individuals or groups, and to carry out network interventions with other social, educational, and health services. Professionals emphasize the need to consolidate the ability to carry out health and social care activities remotely across all services and to formalize telemedicine, with all necessary attention given to IT security. It is necessary, therefore, that rapid investment be made in the deployment of technological resources, which are currently severely lacking in many services, for the appropriate performance of telemedicine activities, as well as training in this regard, considering new organizational models for the delivery of activities that new technologies also impose in the health and social health field.

Professionals emphasized the need to pay special attention to the planning of integrated responses between hospital and territorial facilities, currently lacking in many regional realities, especially in the south and island areas. Integrated responses, specifically aimed at the health needs of the developmental age, are aimed at overcoming inhomogeneity and fragmentation, guaranteeing effective access to the necessary care in the specialized services of child and adolescent neuropsychiatry and developmental rehabilitation, psychological services, ensuring care during and after hospitalization, and acting, also, to counteract the elements of vulnerability with adequate support in social and targeted prevention actions, as well as improve the resilience of caregivers. Household resilience has been cited as a protective factor, so it is important that measures be developed to support it. While some professionals have exhibited a proactive attitude in response to the emergency, it is necessary to implement continuous and coordinated structural and operational actions directed at the prevention and monitoring of neurodevelopmental disorders of persons under the age of 18.

Professionals emphasize how the pandemic exacerbated existing social, economic, and cultural inequalities, and how this was a determinant of the impact on neurodevelopment and mental health. Aged children who were included in a system of social network and organized services and/or who benefited from the proximity of family and/or community were able to activate internal resources and strategies, while those who were already experiencing conditions of fragility, or in school transition, such as pre-adolescents and adolescents, experienced an aggravation of pre-existing discomforts or disorders and, in many cases, even the onset of new problems.

Children’s needs and wishes on policy and institutional decisions must not go unheard. It is essential that children and youth be involved in the choices and be actors in this process. In all contexts, the importance of the group must be considered, not only for the achievement of learning goals, but more generally for the entire growth of children and youth. In the group, characterized by the centrality of emotional and relational processes, children and adolescents manifest their needs, experiment with their abilities, and develop their identity in a continuous exchange with peers, educators, and teachers. To support this process, the creation of a climate of trust among all the components of the group is a fundamental condition so that everyone can feel at the center of the educational process and free to express themselves. Support for psycho-social development in different age groups is essential, promoting activities aimed at listening and observation, recognition and management of emotions, cooperation, and recognition and development of personal resources. The arrangement of activities should be aimed at reinforcing positive role models and promoting resilience and well-being. Supporting a sense of self-efficacy can be especially important in times of fear and uncertainty. To this end, it is helpful to promote experiences and activities in which children and adolescents can play an active role in considering themselves, their families, and their communities.

The added values of this research are the following: (1) being of national significance since it involves experts and professionals from the various parts of the country, both rural and urban; (2) the research is entirely supervised by a Scientific Committee; (3) having a plural character, since it involves experts from different disciplines, qualified representatives of the scientific, academic, and psychosocial professions, and belonging to varied organizational realities; (4) being promoted on a central level of governance by the AGIA, ISS, and Ministry of Education; (5) having enabled the drafting of policies addressed to the central and regional government and institutions.

The limitations of this evidence-gathering work are mainly two-fold: (1) the data must be interpreted considering the rapid development of the pandemic, the interventions for its containment, and the issues related to the mental health of minors of age. In fact, given the rapidly developing epidemiological situation, new phases of the pandemic continue to emerge, and new difficulties related to the mental health of minors may arise, transform, or exacerbate; (2) the data are descriptive and cannot be measured numerically because they derive from a qualitative data collection method and must therefore be interpreted with caution [35].

## 5. Conclusions

The most impactful suggestion among professionals is related to prevention and continuous monitoring in the areas of neurodevelopment and mental health. In this direction, economic and research programming specifically aimed at prevention is needed. Also needed is a strengthening of preventive work and early contact with children, adolescents, and parents to work before the onset of distress, with reference to those who are most vulnerable or exposed to risks. There is a need to value neurodevelopmental and mental health promotion and focus on resource enhancement rather than overt fragilities. Data collection and scientific comparison of the emerging evidence is identified as a foundational tool for achieving the set goals.

Following the research, the Italian Authority for Children and Adolescents made recommendations to the National Government, local Governments, and relevant institutions involved in the protection and care of children and adolescents’ rights, in order to guide their decisions and policies and to ensure that the rights of children and young people are guaranteed regardless of their personal, family, and social background, their origin, or geographical provenance. In particular, the Authority recommended an increase in funds for mental health care and prevention and an increase in professionals with specific skills and training, as well as the implementation of synergic action involving therapeutic and health services, schools, the third sector, and the local actors, in order to ensure continuity of care and accompaniment paths. The construction of a network must take place in a stable and continuous manner and must involve children and young people, encouraging their participation.

## Figures and Tables

**Table 1:** Macro-theme 1: Perceptions, experiences, and factors that are negative and those that are positive and to be enhanced as a result of the pandemic.

Negative Experiences/Factors	Positive Experiences/Factors
Lack of political attention to neurodevelopment and mental health of minors	Quick reactivation of many services, both for remote and in-person activities
Lack of a strategic direction at the national level	Development of remote management of activities, not only healthcare-related
Inconsistent responses depending on territorial and/or organizational contexts	Development of spontaneous networks of exchange among professionals and services
Increase in requests for help not met by services	Development of innovative activities and integrated care pathways between in-person activities and teleassistance
Exacerbation of pre-existing resources and organizational issues in services	Outdoor activities, support from local associations, non-profit, volunteering
Resorting to private services/operators	Perception of active dialogue and collaboration between family and school/health and social services in certain contexts
Loneliness in facing the emergency crisis	Greater involvement and appreciation of services by families already in care before the pandemic

**Table 2:** Macro-theme 2: Perceptions, experiences, and factors negative and to be enhanced as a result of the pandemic.

Negative Experiences/Factors	Positive Experiences/Factors
Greater risk in situations of vulnerability (people with disabilities, migrants, socio-economic fragility, violence, and abuse)	Improvement in the relationship between parents and young children
Greater risk in certain age groups (pre-adolescents and adolescents, especially those in a phase of school transition)	Volunteering opportunities
Worsening of pre-existing neurodevelopmental disorders	Reduction of stigma
Increase in disturbances, symptoms, and conditions of neuropsychiatric fragility	Greater ease in asking for help

**Table 3:** Endogenous and exogenous risk and resilience factors.

Endogenous Factors	Exogenous Factors
Risk factors	
Experiences of isolation, serious illness, and/or the death of one or more family members	Lack of a systemic approach (lack of coordination between social, health, and educational networks)
Complex family situations (such as parents’ separation, absence or overprotection of adult reference figures, parents’ work overload or high-risk COVID jobs)	Lack of a sufficiently effective network of social, health, and educational services (such as child neuropsychiatry services, psychology, school, and social services)
Pre-existing psychological and neuropsychiatric problems	Inadequacy of reception and care systems
Stress related to the demand for high academic performance	Extended school closures
Difficulty in managing daily routines	Constant perception of uncertainty and distrust in institutions
Inadequate and/or excessive use of technological devices for educational activities and social relationships (such as excessive use of social networks)	Lack of green areas and prolonged closure of places of aggregation and/or socialization
Lack of knowledge of the Italian language by migrants and their families	Confusion generated by media communication
Lack or inadequacy of computer resources	Lack or inadequacy of computer resources
Episodes of violence against minors and assisted violence	Socio-cultural and economic fragility (such as precarious job positions or loss of parents’ jobs)
Protective factors	
Resilience and family balance in emergency management	Presence or strengthening of social, health, and educational networks capable of implementing synergistic collaborations and alternative strategies
Parental support in carrying out distance learning. For students with disabilities and special educational needs, possibility of small in-person groups	Stimulating school and extracurricular activities (such as workshops, linguistic-cultural facilitation, enhancement of social and sports activities)
Adaptability of migrants and those living in communities	Social inclusion projects for migrants
Availability of technological devices for school and social activities	Availability of technological devices for school and social activities
Green areas, easily accessible open spaces	Spacious living spaces, with the possibility of open spaces
	Training of social and health workers, teachers, and students, and digital literacy of parents

**Table 4:** Prevention strategies.

Prevention Strategies
“Networking” among services and implementing stable teams
Foster homogeneous responses throughout the country
Share good practices
Increase health, social, and social work staff in multidisciplinary teams
Increase inpatient beds in child and adolescent neuropsychiatry wards
Dedicate funds and research for prevention and continuous monitoring in the areas of neurodevelopment and mental health
Systematically use telemedicine and telehealth
Increase the consideration of inequalities and social determinants in clinical practice
Stimulate and strengthen the focus on neurodevelopment and a bio-psycho-social growth model of children and young people
Enhance spaces of integration and socialization for children and adolescents
Increase awareness and use of advocacy tools
Promote actions to support parenting

**Table 5:** Strategies for prevention suggested by professionals in relation to the thematic insights.

Thematic Insights
School
Give schools a central role in promoting and monitoring the neurodevelopment and mental health of children and adolescents
Allocate more systemic investments towards neurodevelopment and mental health
Include a psychologist affiliated with school psychology services within family clinics in all schools
Support ongoing teacher training (e.g., use of digital tools, moving beyond frontal teaching)
Value the tool of educational pacts
Encourage peer education-based training strategies
Unaccompanied foreign minors
To define more standardized and uniform procedures, and disseminate them in all territories
Addiction
Allocate funds and research for the prevention of addictions and promotion of mental health

## Data Availability

Not applicable.
